# GPKOW is essential for pre-mRNA splicing *in vitro* and suppresses splicing defect caused by dominant-negative DHX16 mutation *in vivo*

**DOI:** 10.1042/BSR20140142

**Published:** 2014-12-12

**Authors:** Shengbing Zang, Ting-Yu Lin, Xinji Chen, Marieta Gencheva, Alain N. S. Newo, Lixin Yang, Daniel Rossi, Jianda Hu, Shwu-Bin Lin, Aimin Huang, Ren-Jang Lin

**Affiliations:** *Department of Molecular and Cellular Biology, Beckman Research Institute of the City of Hope, Duarte, California, U.S.A.; †Department of Pathology, School of Basic Medical Sciences, Fujian Medical University, Fuzhou, China; ‡Department of Clinical Laboratory Sciences and Medical Biotechnology, National Taiwan University, Taipei, Taiwan; §Fujian Institute of Hematology, Union Hospital of Fujian Medical University, Fuzhou, China

**Keywords:** DExD/H-box protein, G-patch domain, KOW domain, RNA helicase, spliceosome, splicing factor, DExD/H, aspartate–glutamate-x-aspartate/histidine, EGFP, enhanced green fluorescent protein, EMSA, electrophoretic mobility shift assay, G-patch, glycine-rich domain, HEK-293T cell, HEK-293 cells expressing the large T-antigen of SV40 (simian virus 40), KOW domain, Kyrpides, Ouzounis and Woese domain, NP40, Nonidet P40, snRNA, small nuclear RNA

## Abstract

Human GPKOW [G-patch (glycine-rich) domain and KOW (Kyrpides, Ouzounis and Woese) domain] protein contains a G-patch domain and two KOW domains, and is a homologue of *Arabidopsis* MOS2 and *Saccharomyces* Spp2 protein. GPKOW is found in the human spliceosome, but its role in pre-mRNA splicing remains to be elucidated. In this report, we showed that GPKOW interacted directly with the DHX16/hPRP2 and with RNA. Immuno-depletion of GPKOW from HeLa nuclear extracts resulted in an inactive spliceosome that still bound DHX16. Adding back recombinant GPKOW restored splicing to the depleted extract. *In vivo*, overexpression of GPKOW partially suppressed the splicing defect observed in dominant-negative DHX16 mutant expressing cells. Mutations at the G-patch domain greatly diminished the GPKOW–DHX16 interaction; however, the mutant was active in splicing and was able to suppress splicing defect. Mutations at the KOW1 domain slightly altered the GPKOW–RNA interaction, but the mutant was less functional *in vitro* and *in vivo*. Our results indicated that GPKOW can functionally impact DHX16 but that interaction between the proteins is not required for this activity.

## Abstract 2

Using biochemical, mutation, and cellular analyses, we characterized important domains involved in the functionality of a RNA-binding protein in RNA splicing. We also showed the similarity and difference between yeast and human counterparts.

## INTRODUCTION

Pre-mRNA splicing removes introns from messenger RNA precursors and the reaction occurs in the spliceosome, which contains five snRNAs (small nuclear RNAs) and more than a hundred proteins [1, 2]. Dynamic RNA–RNA, RNA–protein and protein–protein interactions among the spliceosomal components are hallmarks of pre-mRNA splicing [3, 4]. At least eight DExD/H (aspartate–glutamate-x-aspartate/histidine)-box proteins are required for pre-mRNA splicing and many of them, in conjunction with RNA-binding proteins, are long thought to be among the driving forces behind the remodelling of interactions among spliceosomal proteins and RNAs [5, 6].

Budding yeast Prp2 and human DHX16 are orthologous DExH-box proteins required for advancing an activated spliceosome to a catalytic one [7, 8]. The ATPase activity of Prp2 is essential for its splicing function [9, 10] and the ATP hydrolysis activity of Prp2 is stimulated by RNA [11]. Chemically modified U6 snRNA that does not coordinate a catalytically important magnesium stalls Prp2 on the spliceosome [12, 13]. It is speculated that a spliceosomal RNA triggers the ATP hydrolysis by Prp2 within the spliceosome [14, 15]; however, it is still not clear which RNA is most critical for this function. Moreover, the involvement of RNA-binding protein in this process is largely unknown, even though several spliceosomal proteins working around Prp2 have been identified [16–20].

To further understand the function of DHX16/hPRP2, we used a yeast two-hybrid screen to search for interacting proteins. Human GPKOW [G-patch (glycine-rich) domain and KOW (Kyrpides, Ouzounis and Woese) domain] protein was identified in the screen. The G-patch domain in GPKOW is very similar to that of yeast Spp2, a spliceosomal protein that interacts with yeast Prp2 [16, 21]. Since the two-hybrid interaction between DHX16 and GPKOW has recently been reported and GPKOW is found in spliceosome preparations [22], it is possible that GPKOW is a co-factor for DHX16 function in the spliceosome [22, 23].

In this study, we investigated the role of GPKOW in splicing by characterizing the wild-type and two mutant proteins. We showed that GPKOW directly interacts with DHX16 and with RNA. Mutations in the G-patch domain impaired the GPKOW–DHX16 interaction; however, the G-patch mutation did not abolish the splicing activity of GPKOW. Mutations in the first (N-terminal) KOW domain did not affect the protein interaction, but the KOW1 mutation weakened the splicing activity of GPKOW. In addition, we showed that GPKOW suppressed DHX16 mutant-induced splicing defect *in vivo*.

## MATERIALS AND METHODS

### Plasmid construction

pET28a^(+)^-GPKOW was constructed by inserting a PCR amplicon of GPKOW with primers containing BamHI and XhoI sites and the construct was verified by sequencing. The recombinant protein has a His_6_-tag at N-terminus. The GK/AA and GW/AA mutations in GPKOW were introduced using a site-directed mutagenesis kit (Stratagene). EGFP (enhanced green fluorescent protein)-fused GPKOW proteins, including WT, GK/AA and GW/AA, were made by inserting the BamHI/XhoI fragment of GPKOW into the Bgl II and Sal I sites of pEGFP-C1 (Clontech). The fusion protein has EGFP at its N-terminus. Construction of Flag-DHX16 has been described [8]. Oligo sequences are listed in Supplementary Table S1.

### Recombinant protein expression and purification

*Escherichia coli* Rosetta (DE3)pLysS cells were transformed with pET28a(+) constructs and induced with 1 mM IPTG (isopropyl β-D-thiogalactoside) at 37 °C for 5 h. Cells were then resuspended in 50 mM NaH_2_PO_4_, pH 7.0, 300 mM NaCl, 0.1% (v/v) Triton X-100, 100 μg/ml lysozyme and lysed by freezing–thawing and passing through a 25-gauge needle. The extract was cleared by centrifugation and then mixed with TALON® Metal Affinity Resins (Clontech) for 1 h in a chromatography column at 4 °C. The resins were then washed with the same buffer plus 10 mM imidazole. His_6_-tagged protein was eluted in buffer containing 150 mM imidazole. Fractions containing the recombinant protein were dialysed against 20 mM Hepes-KOH, pH 7.9, 50 mM KCl, 10% (v/v) glycerol, 0.5 mM PMSF and 0.5 mM β-mercaptoethanol. The cobalt fractions were loaded onto a poly(U) Sepharose (Amersham Biosciences) column equilibrated with the buffer containing 0.01% (v/v) NP40 (Nonidet P40). GPKOW protein was step-eluted with increasing KCl from 100, 150, 200 to 250 mM. Protein purity was verified by SDS–PAGE with Coomassie Brilliant Blue staining.

### Anti-GPKOW and anti-peptide antibodies production

Antiserum against purified recombinant His_6_–GPKOW protein (anti-GPKOW) was raised in rabbit by certified personnel at the City of Hope Animal Resources Center in accordance with protocol #93023 as approved by the Institutional Animal Care and Use Committee. Affinity-purified polyclonal rabbit antibodies against GPKOW peptides were made by GenScript Corporation. Two peptides were used: NGHRRQPPARPPGPC from the N-terminal portion (peptide1) and RPDEEQEKDKEDQPC from the region between G-patch and KOW1 (peptide2). We found anti-peptide1 reacted with the GPKOW protein, but anti-peptide2 did not.

### Cells and transfection

Cells were cultured in DMEM (Dulbecco's modified eagle medium; Irvine Scientific) supplemented with 10% (v/v) FBS (HyClone) in 5% (v/v) CO_2_ at 37 °C. Typically, cells (8×10^5^) were transfected with plasmid DNA (3 μg) using Lipofectamine 2000 (Invitrogen) and cultured for 48 h before assayed. When needed, cells were also co-transfected with a minigene reporter plasmid (300 ng) [24].

### Cell lysate preparation, Western and Northwestern blotting

Cells were lysed in hypotonic buffer (10 mM Hepes/pH 7.9, 1.5 mM MgCl_2_, 10 mM KCl, 0.2 mM PMSF and 1 mM DTT) containing 0.6% NP40, and centrifuged at 12000 ***g*** for 20 s to obtain the nuclear pellet. The supernatant was collected as the cytosolic fraction. The nuclear pellet was lysed in RIPA buffer [10 mM sodium phosphate/pH 7.2, 150 mM NaCl, 1% (w/v) sodium deoxycholate, 1% NP40, 0.1% (w/v) SDS, 2 mM EDTA, 1 mM DTT, 1 mM PMSF and 100 units/ml benzonase] on ice, and centrifuged to collect the nuclear fraction. For total cellular protein, cells were lysed directly in RIPA buffer and the lysate was collected by centrifugation. For Western, protein lysates were resolved in SDS–PAGE gel, transferred to PVDF membrane, incubated with primary antibodies, reacted with IRDye680CW-labelled goat anti-rabbit or IRDye800CW-labelled goat anti-mouse secondary antibodies and detected by Odyssey Classic scanner (Licor). Antibodies against DHX16 [8], topoisomerase II (Sigma), tubulin (Sigma) and GAPDH (Ambion) were used. For Northwestern, proteins were electrophorezed, transferred to PVDF membrane and visualized by staining with Ponceau S. The membrane was conditioned in NW buffer [10 mM Tris–HCl/pH 7.5, 50 mM NaCl, 1 mM EDTA, 0.02% (w/v) Ficoll 400 and 0.02% (w/v) polyvinylpyrolidone-40] containing 1% (w/v) BSA, incubated with ^32^P-labelled RNA in the presence of tRNA (50 μg/ml), exposed in PhosphorImager (Molecular Dynamics) and analysed using ImageQuant TL software.

### Protein interaction assay by His-tagged pull-down or immunoprecipitation

For His-tagged pull down, 100 μl *E. coli* lysate containing His_6_-tagged protein was incubated with 10 μl Co^+^-resin (Clontech), washed in the buffer containing 10 mM imidazole and 0.05% Triton X-100 and equilibrated in the binding buffer (50 mM Na-phosphate/pH 7.0, 300 mM NaCl, 5% glycerol, 0.05% Triton-X100, 0.5 mM β-mercaptoethanol, 0.5 mM PMSF and 1.5 mM MgCl_2_). Flag-DHX16-expressing HEK-293T cell [HEK-293 cells expressing the large T-antigen of SV40 (simian virus 40)] lysates (10 μl) were added, incubated for 2 h, and the beads were then washed in the binding buffer containing 0.3% Triton X-100. Bound proteins were eluted by boiling in SDS–PAGE loading buffer. For immunoprecipitation, cells were lysed by sonication in 20 mM Hepes-KOH/pH 7.9, 100 mM KCl, 0.2 mM EDTA, 10% glycerol, 1 mM DTT and 1 mM PMSF. Antibodies were bound to 5 μl Protein A Sepharose beads (GE healthcare) in 20 mM Tris–HCl/pH 7.5, 0.5 M NaCl, 0.05% NP40 and 2% BSA. Protein lysates (10 μg) in TBS/NP40 (20 mM Tris–HCl/pH 7.5, 0.15 M NaCl and 0.05% NP40) was added and incubated at 4 °C for 2 h. The immunoprecipitated proteins were recovered by boiling the beads in SDS–PAGE loading buffer. For digestion of RNA prior to immunoprecipitation, lysates were incubated with 5 μg/mL RNase A (Ambion) for 15 min at 37 °C.

### Electrophoretic mobility shift assay (EMSA) for RNA-binding activity

The recombinant proteins were incubated with ^32^P-labelled RNA at 4 °C in binding buffer (20 mM Hepes-KOH/pH7.9, 150 mM KCl, 2 mM MgCl_2_, 0.2 mM DTT, 2 mM DTT and 0.02% NP40). The reaction mixture was electrophorezed on 5% (w/v) polyacrylamide non-denaturing gels. The gels were dried and exposed to PhosphorImager.

### *In vitro* transcription, splicing and spliceosome assays

^32^P-labelled pre-mRNA from pRG1, ^32^P-labelled snRNAs, ^32^P-labelled antisense RNA of U1, U2, U4, U5 and U6 were prepared as previously described in [8] using [α-32P]UTP and T7 or SP6 polymerase (NEB), and isolated by passing through a G-50 column (Ambion). RNA was then ethanol precipitated in the presence of 10 μg glycogen and resuspended in water. Nuclear splicing extracts were prepared [8, 25], and *in vitro* splicing reactions were performed as described in [8, 26]. The splicing efficiency was defined as the ratio of the spliced products to the total RNA species. Analysis of spliceosomal complexes was performed as previously described in [8, 27]. Briefly, splicing reactions were stopped by adding 0.1 volume of 10X heparin loading dye [6.5 mg/ml heparin sulphate, 40% glycerol, 0.5% (w/v) bromophenol blue and 0.5% (w/v) xylene cyanol in 1X TBE (tris/borate/EDTA)] and run on 2% Seakem agarose (Lonza) gel in 50 mM Tris/50 mM glycine.

### Immunodepletion of GPKOW from nuclear extract

Anti-GPKOW antiserum (50 μl), mixed with 300 μl TBS (20 mM Tris–HCl, pH 7.5 and 500 mM NaCl) containing 0.05% NP40 and 2% BSA, was incubated with 60 μl of 50% protein A-Sepharose beads for 1 h at 4 °C with head-over-tail rotation. The beads were then washed with TBS plus 0.05% NP40 and with splicing extract buffer (buffer D: 20 mM Hepes-KOH, pH 7.9, 20% glycerol, 100 mM KCl, 0.2 mM EDTA, 0.5 mM PMSF and 1 mM DTT). The beads were then incubated with 100 μl of HeLa S3 nuclear extract for 2 h at 4 °C. The immunodepleted extract (∆GPKOW) was frozen in liquid nitrogen and stored at −80 °C until used. The degree of GPKOW depletion was verified by western blot analysis.

### Immunoprecipitation after splicing reaction

Antibody against GPKOW or serum was incubated with Protein A Sepharose in IP buffer (20 mM Tris–HCl/pH 7.5, 0.15 M NaCl and 0.05% NP40). Heparin was added to the splicing reaction (25 μl) at 0.2 μg/μl, and the mixture was incubated with Protein A beads (5 μl) coated with antibodies in the IP buffer. After an addition of 200 μl of 0.3 M NaOAc/pH5.2, 0.1% (w/v) SDS, RNA was isolated by phenol extraction and ethanol precipitation.

### Glycerol gradient sedimentation

The HeLa nuclear extract was loaded on a glycerol gradient (20 mM Hepes-KOH/pH7.9, 50 mM KCl, 1.5 mM MgCl_2_, 1 mM DTT and 10–30% glycerol) and centrifuged at 120000 ***g*** for 3 h in an SW41 rotor (Beckman-Coulter) [28]. The fractions were recovered from top to bottom. RNA was isolated and analysed by Northern blotting with ^32^P-labelled RNA complementary to U1, U2, U4, U5 and U6 snRNAs [24]. Protein was isolated and immunoblotted as described.

### RT–PCR (reverse transcription–PCR)

Total RNA was extracted using TRIzol (Invitrogen) and treated with DNase I (1 unit for 1 μg total RNA) at 37 °C for 30 min. DNase I-treated RNA (300 ng) was reverse transcribed to cDNA with random hexamers (NEB) using SuperScriptIII (Invitrogen), and 1/20 of the cDNA product was used for PCR amplification (Sigma). The primer sequences are listed in supplementary Table S1.

### Microscopy

Cells seeded on cover slides on a 6-well plate were transfected with plasmids for 24 h. Cells were then fixed with 4% (w/v) paraformaldehyde, permeabilized in PBS containing 0.3% Triton X-100, and stained with 300 nM DAPI (Sigma). The images were obtained on an Olympus IX81 inverted automatic microscope.

## RESULTS

### Protein interaction between DHX16 and GPKOW

Yeast genetic suppression was instrumental in identifying Spp2 as a co-factor for Prp2 in yeast pre-mRNA splicing [21], and that interaction was independently identified by a yeast two-hybrid screen [16]. We therefore used yeast two-hybrid screen to look for potential protein co-factors for hPRP2/DHX16. Three splicing factors were identified: SLU7, RBM10 and GPKOW (Supplementary Figure S1). We chose to study GPKOW for it contains a G-patch domain and is homologous to yeast Spp2. The DHX16–GPKOW interaction was confirmed by reconstructing a GAL4–GPKOW fusion and it indeed showed interaction with a GBKT7–DHX16 construct (results not shown).

**Figure 1:**
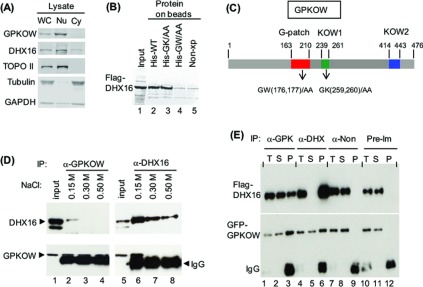
Interaction between GPKOW and DHX16 (**A**) Immunoblots showing GPKOW is in the nucleus. Whole-cell (WC), nuclear (Nu), and cytosolic (Cy) lysates from HEK-293 cells were immunoblotted with antibodies as indicated. GPKOW was probed with the anti-peptide 1 antibody. Topoisomerase II (TOPO II) is nuclear, while tubulin and GAPDH are cytosolic. (**B**) An immunoblot showing a pull-down of Flag-DHX16 by His_6_–GPKOW. Flag-DHX16-expressing HEK-293T cell lysate was incubated with cobalt beads containing bacterially expressed His_6_-tagged GPKOW (His–WT; lane 2) or control bacterial lysate (Non-xp; lane 5). Bound proteins were immunoblotted with anti-Flag. Cobalt beads containing bacterially expressed His_6_-GK/AA (lane 3) or His_6_-GW/AA (lane 4) were also used. Input was the DHX16-transfected HEK-293T lysate (lane 1). (**C**) A diagram depicting the domain arrangement in GPKOW: red, G-patch; green, KOW1; blue, KOW2. Numbers represent the amino acid position from the amino-terminus; the GW/AA and GK/AA mutants are indicated. (**D**) Immunoblots showing co-immunoprecipitation of endogenous DHX16 and GPKOW in HeLa nuclear extracts. Splicing competent extracts were incubated with beads containing anti-GPKOW or anti-DHX16 antibodies in the presence of various NaCl concentrations as indicated. Protein from the immunoprecipitates (IP) was immunoblotted with anti-DHX16 (upper panel) or anti-GPKOW (lower panel). Input was the mixture containing the nuclear extract and the beads. (**E**) Immunoblots showing co-immunoprecipitation of ectopically expressed Flag-DHX16 and GFP–GPKOW in HEK-293T lysates. The lysates were immunoprecipitated with anti-peptide1 (α-GPK), anti-DHX16 (α-DHX), non-reactive anti-peptide2 (α-Non) or pre-immune (Pre-Im) antibodies: T, the mixture containing the lysate and the beads; S, supernatant; P, pellet. Protein was immunoblotted with anti-DHX16 (upper panel) or anti-GPKOW (lower panel).

Polyclonal antibodies against an N-terminal peptide or against purified recombinant His_6_-tagged GPKOW were generated. By probing nuclear and cytosolic protein fractions from HEK-293 cells with the antibodies, we found that GPKOW was present almost exclusively in the nuclear fraction, while DHX16/hPRP2 was mostly in the nuclear fraction (Figure 1A).

We performed a pull-down assay to investigate the interaction of GPKOW and DHX16. Bacterially expressed His_6_–GPKOW protein was purified on Co^2+^-resin and incubated with lysates isolated from Flag-DHX16-expressing HEK-293T cells; Flag-DHX16 was pulled down by His_6_-WT GPKOW (Figure 1B, lane 2). We then used this assay to determine which part of GPKOW is important for the DHX16-interaction. We constructed two double-alanine mutants of GPKOW; GW(176, 177) are conserved amino acid residues in the G-patch domain and GK(259, 260) are conserved residues in the KOW domain (Figure 1C; Supplementary Figure S2). Flag-DHX16 was pull downed by the His_6_–GK/AA protein (Figure 1B, lane 3), but not by the His_6_–GW/AA (lane 4). This result indicated that the G-patch domain on GPKOW is important for the DHX16 interaction, an observation analogous to the yeast Prp2–Spp2 interaction [16].

We then assayed the endogenous DHX16–GPKOW interaction in HeLa nuclear extracts by co-immunoprecipitation. Nuclear extracts were incubated with anti-GPKOW or anti-DHX16 antibodies bound to protein A beads (Figure 1D). DHX16 was co-immunoprecipitated with GPKOW (lane 2) and GPKOW with DHX16 (lane 6) in buffer containing 0.15 M NaCl. The interaction was disrupted when NaCl concentration was increased to 0.3 or 0.5 M (lanes, 3, 4, 7 and 8). Because GPKOW migrated very close to the IgG, we further clarified the interaction by transient co-expression of Flag-DHX16 and GFP–GPKOW in HEK-293T cells. Again, Flag-DHX16 was immunoprecipitated with anti-GPKOW (Figure 1E, lane 3) and GFP–GPKOW with anti-DHX16 (lane 6). The pre-immune serum or the non-reactive anti-peptide2 antibody did not bring down either GPKOW or DHX16 (lanes 12 and 9). Thus, DHX16 and GPKOW indeed interact with each other in nuclear extracts. The co-immunoprecipitation of Flag-DHX16 and GFP–GPKOW was not affected by treating the lysate with RNase A (results not shown). Taken together, the results indicate that DHX16 and GPKOW interact via protein–protein contact and the G-patch domain is important for that interaction.

### GPKOW binds directly to RNA

**Figure 2:**
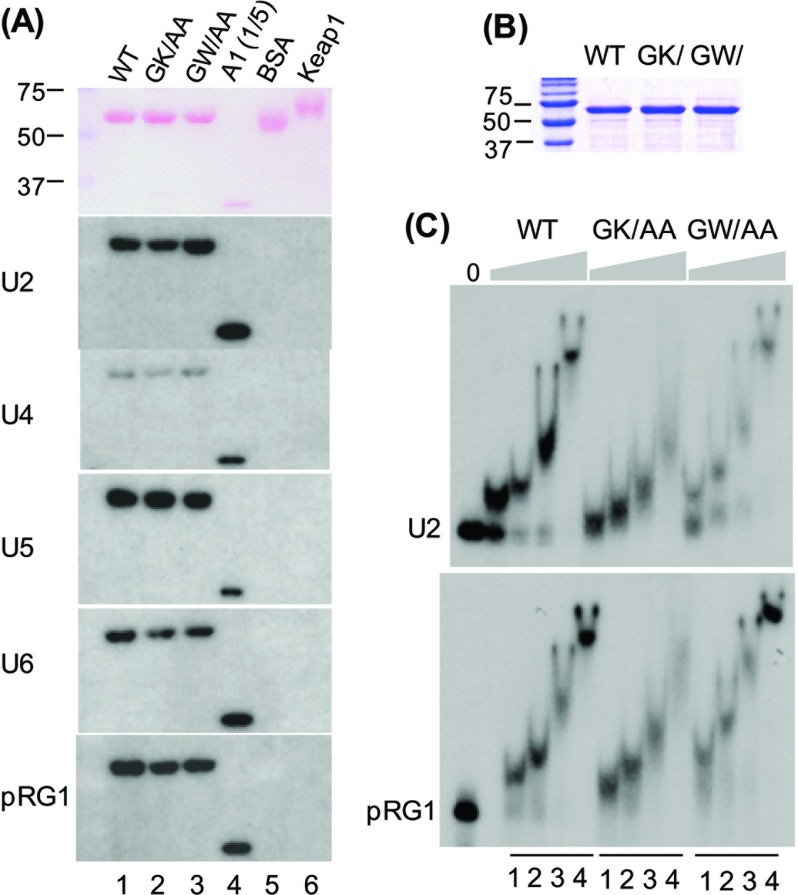
RNA binding by GPKOW (**A**) Northwestern blots showing direct binding of RNA to GPKOW. Recombinant His_6_-tagged GPKOW (WT), GPKOW mutants, hnRNP A1 and Keap1 proteins were purified from *E. coli*, electrophorezed on SDS-polyacrylamide gels, transferred to PVDF membranes and stained with ponceau S (top panel). The membranes were incubated with ^32^P-labelled RNA as indicated and autoradiographed. An equal amount of protein was used in all samples except that 1/5 of that amount was used in His_6_-tagged hnRNP A1. (**B**) A Coomassie Brilliant Blue-stained gel showing equal amounts of the recombinant His_6_-tagged GPKOW (WT), GK/AA (GK/) and GW/AA (GW/) used in (C). Proteins were purified by a cobalt affinity column followed by a poly(U) column. (**C**) Electrophoresis mobility shift assay showing the complexes formed between the protein and the ^32^P-labelled U2 snRNA or the pRG1 RNA. Four protein concentrations were used (1–4 in each case): 0.25, 0.5, 1.0 and 2.0 μM. The complexes were resolved on 5% polyacrylamide non-denaturing gels.

It has been reported that cellular RNA can be immunoprecipitated with GPKOW upon UV cross-linking; however, it is not clear whether the interaction is direct [29]. Here we used Northwestern and gel shift assays to biochemically characterize GPKOW–RNA interaction. In a Northwestern assay, we electrophorezed purified recombinant GPKOW proteins on SDS–PAGE gels (Figure 2A, top panel); upon renaturation, immobilized proteins were incubated for direct binding with ^32^P-labelled RNA (bottom panels). Wild-type GPKOW and the control hnRNP A1 bound U2, U4, U5 and U6 snRNAs, as well as pre-mRNA transcribed from pRG1 (Figure 2A, lanes 1 and 4); while no RNA binding occurred with BSA or with signalling protein Keap1 (lanes 5 and 6). The GK/AA and GW/AA mutant proteins also bound RNA with similar efficiencies (Figure 2A, lanes 2 and 3). By performing EMSA using purified recombinant proteins (Figure 2B) and U2 or pRG1 RNA, larger, slow-migrating RNA–protein complexes were formed with increasing amounts of proteins (Figure 2C). Under the same conditions, the GK/AA protein formed smaller complexes than the WT protein, while the GW/AA protein formed larger complexes than the WT (Figure 2C). The reason for these differences was currently not clear, since they could be caused by a change of RNA–protein interaction, protein–protein interaction or both. Nevertheless, the result indicated that the GPKOW protein can directly bind to RNA.

### GPKOW is assembled into the spliceosome

**Figure 3:**
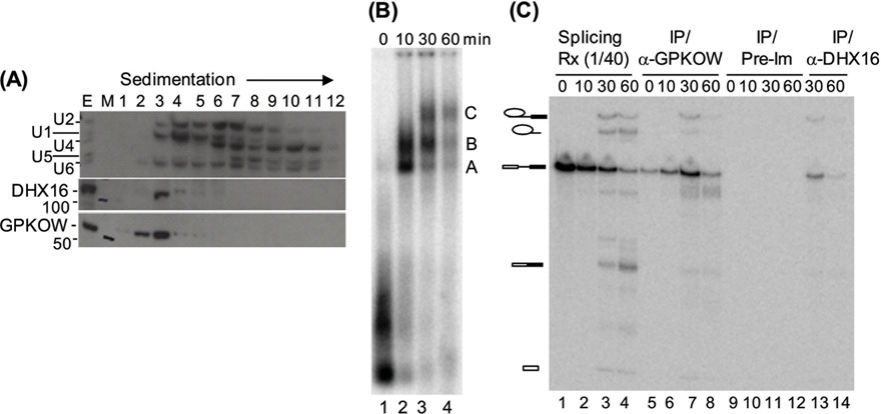
Incorporation of GPKOW into the spliceosome (**A**) Northern and immunoblots of glycerol gradient fractions of HeLa nuclear extracts. Extracts were sedimented through a glycerol gradient and fractions were collected from top (Fraction 1) to bottom (Fraction 12). RNA was analysed by Northern blotting (upper panel) with ^32^P-labelled RNA complementary to U1, U2, U4, U5 and U6 snRNAs. Protein was immunoblotted with anti-DHX16 (middle panel) and anti-GPKOW (lower panel). E, the nuclear extract; M, protein size markers and 100 and 50 are marks of the 100 and 50 kDa protein markers, respectively. (**B**) A non-denaturing gel showing *in vitro* spliceosome assembly. Splicing reactions were carried out for 0, 10, 30 or 60 min and the reaction mixture was electrophorezed on an agarose gel. Splicing complexes A, B and C are indicated. (**C**) A denaturing gel showing the ^32^P-labelled RNA co-immunoprecipitated using anti-GPKOW, anti-DHX16, or the pre-immune serum (lanes 5–14). Lanes 1–4 are 1/40 of the RNA from the splicing reaction. Drawings from top to bottom: lariat-exon 2, lariat intron, pre-mRNA, spliced RNA and exon 1.

We sedimented splicing extracts in a glycerol gradient and analysed the distribution of snRNAs (by Northern), DHX16 and GPKOW (by immunoblotting) among the gradient fractions (Figure 3A). Although the majority of both proteins appeared not to be in free, monomeric configuration (for those sedimented in fraction 3 that contained snRNPs), little of either protein was associated with a large complex without spliceosome assembly. Splicing reactions were carried out using ^32^P-labelled pRG1 RNA and analysed by native gels for splicing complexes A, B and C (Figure 3B). To investigate whether and when GPKOW is incorporated into a splicing complex, the reaction mixtures were incubated with anti-GPKOW antibodies and the ^32^P-labelled RNA in the immunoprecipitates was resolved in a denaturing gel (Figure 3C). Pre-mRNA and the splicing intermediates (exon 1 and intron–exon 2) were brought down with GPKOW most visibly at the 30-min time point when splicing complexes B and C were formed (Figure 2B, lane 3 and Figure 3C, lane 7). At the same 30-min time point, the pre-mRNA and the intermediate RNAs were associated with DHX16 (Figure 3C, lane 13). This result indicated that GPKOW is assembled into complexes B and C during splicing, similar to its interacting protein DHX16 [8].

### GPKOW is required for splicing

**Figure 4:**
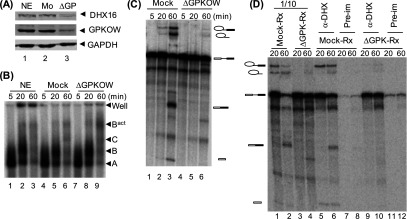
Losing splicing activity in GPKOW-depleted extracts (**A**) Western blots showing immunodepletion of GPKOW from HeLa nuclear extracts. Extracts were incubated with protein A beads pre-treated with preimmune (Mo) or anti-GPKOW (ΔGP) antiserum, and immunoblotted with anti-DHX16 (upper panel), anti-GPKOW (middle panel) or anti-GAPDH (lower panel). (**B**) A non-denaturing gel showing splicing complexes assembled in splicing reactions. Splicing reaction was carried out in HeLa nuclear extracts, mock-, or GPKOW-depleted extracts (ΔGPKOW) for the time indicated. Splicing complexes A, B, B^act^ and C are marked. (**C**) Denaturing RNA gels showing the splicing activity in GPKOW-depleted extracts. Splicing reactions were carried out in mock depleted (lanes 1–3) or GPKOW depleted (lanes 4–6) extracts. (**D**) A denaturing gel showing ^32^P-labelled RNA co-immunoprecipitated with anti-DHX16. After incubation with mock- or GPKOW-depleted extracts, RNA was extracted from splicing mixtures (lanes 1–4; 1/10 of the total amount was analysed) or from immunoprecipitates using anti-DHX16 (lanes 5, 6, 9–10) or pre-immune serum (lanes 7, 8, 11, 12).

To test whether GPKOW is required for splicing, we removed the protein from HeLa nuclear splicing extract by immunodepletion using anti-GPKOW antibodies (Figure 4A). A vast majority of the endogenous GPKOW was removed with a significant amount of DHX16 was also removed due to their interaction (Figure 4A, lane 3). The depleted extract was used in splicing reaction using ^32^P-labelled pre-mRNA, and the splicing complexes assembled in the reaction were analysed on native agarose gels. The kinetics of splicing complex formation in the depleted extract was very similar to the control extracts (Figure 4B); except there was slight accumulation of complex B^act^ in the depleted reaction at 60 min of incubation (lane 9). RNA in the splicing reactions was isolated and analysed on denaturing polyacrylamide gels; splicing occurred in the mock-treated extract, but almost not in the depleted extract (Figure 4C). The lack of splicing was probably not due to the decrease of DHX16 in the depleted extract (Figure 4A), because the pre-mRNA could still be brought down by anti-DHX16 at the 60-min time point (Figure 4D, lane 10) indicating the presence of DHX16 in the spliceosome.

**Figure 5:**
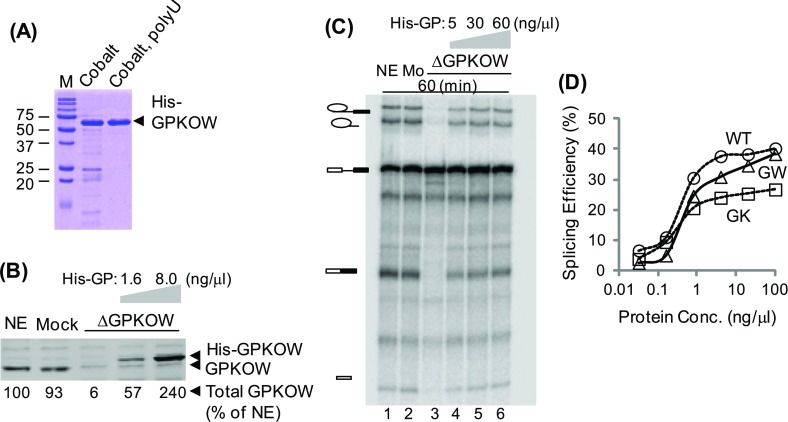
Restoration of splicing activity in GPKOW-depleted extracts by GPKOW (**A**) A Coomassie Brilliant Blue stained gel showing the purity of the recombinant His_6_–GPKOW protein used. (**B**) Western blots showing the adding back of His_6_–GPKOW (at 1.6 and 8.0 ng/μl) to the depleted extracts. (**C**) A denaturing RNA gels showing the splicing activity in GPKOW-depleted extracts with His_6_–GPKOW added back at 5, 30 or 60 ng/μl (lanes 4–6). Reaction time was 60 min. (**D**) Graph summarizing the rescue experiments using GPKOW mutants. Purified His_6_–GPKOW (WT), His_6_–GK/AA (GK) or His_6_–GW/AA (GW) protein (0.032–100 ng/μl) was added to the GPKOW-depleted extracts (Supplementary Figure S3). Splicing efficiency was calculated by comparing spliced products with unspliced pre-mRNA and normalized to the reactions using untreated extracts. Circle, WT; triangle, GW; square, GK.

To test whether GPKOW is needed for splicing, recombinant His_6_–GPKOW protein purified via cobalt-binding and poly(U)-binding (Figure 5A) was added back to the depleted extract (Figure 5B). Splicing was restored to the depleted extracts when His_6_–GPKOW protein was added back (Figure 5C, lanes 4–6), but the activity could not be completely restored (compared with lanes 1 and 2) even with a large excess of His_6_–GPKOW. It is possible that the recombinant protein expressed in *E. coli* was not fully functional, or the incomplete rescue is due to partial removal of DHX16 (or other spliceosomal components) during the immunodepletion (Figure 4A). Nevertheless, the rescue experiment provided strong evidence that GPKOW is required for pre-mRNA splicing *in vitro*.

We used the depleted extracts to assay the *in vitro* splicing activity of GPKOW with the G-patch or KOW1 mutation. Different amounts of purified His_6_–GPKOW proteins (Figure 2B) were added and the splicing reaction was carried out for 60 min (Supplementary Figure S3). Both mutant proteins rescued the depleted extracts almost as efficient as the wild-type, except at high concentrations in which the GK/AA mutant was slightly less effective (Figure 5D). It was somewhat unexpected that the GW/AA mutant, which virtually did not interact with DHX16 in the His-tagged pull-down assay (Figure 1B), rescued the depleted extracts (Figure 5C; Supplementary Figure S3; Figure 5D). Thus, it appeared that the GW-to-AA mutation in the G-patch motif, which abolished GPKOW–DHX16 pairwise interaction, did not impair the splicing activity of GPKOW.

### GPKOW suppresses DHX16 dominant-negative mutation-induced splicing defect

Expression of DHX16/hPRP2 with a dominant-negative mutation impairs cellular pre-mRNA splicing and accumulates intron-containing RNAs [24], which is analogous to the expression of yeast prp2 with similar mutations that results in pre-mRNA accumulation [10, 30]. Overexpressing yeast SPP2 genetically suppresses prp2 mutant alleles [16, 21]. Here we tested whether GPKOW would functionally impact DHX16 mutations *in vivo*.

**Figure 6:**
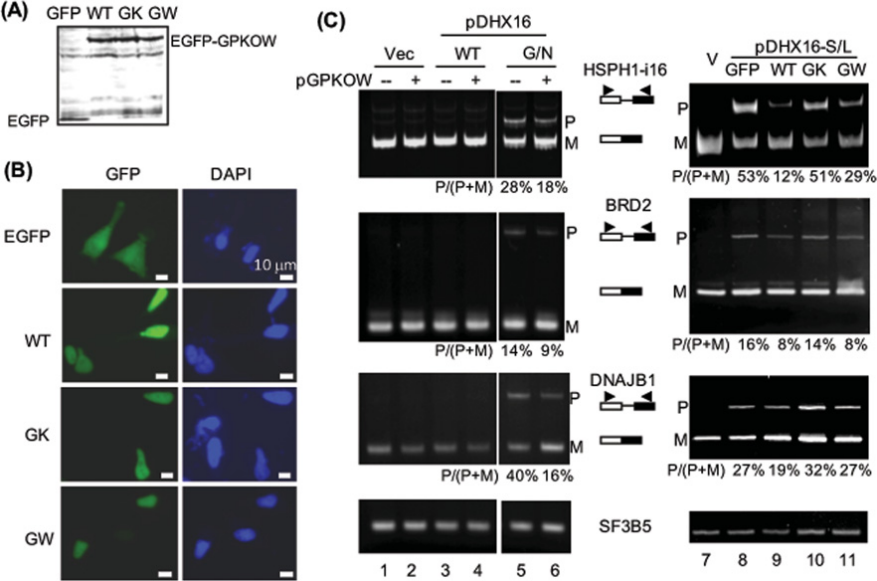
Partial suppression by GPKOW of splicing defect in dominant-negative DHX16 expressing cells (**A**) An immunoblot showing ectopically expressed GFP-fusion protein in HEK-293T cells transfected with pEGFP (GFP), pEGFP-GPKOW (WT), pEGFP-GK/AA (GK) or pEGFP-GW/AA (GW). Protein lysates were probed with anti-GFP antibodies. (**B)** Micrographs showing cellular localization of the GFP-fusion proteins. Transfected cells were observed for green fluorescence (left) and blue DAPI staining (right). (**C**) RT–PCR gels detecting unspliced (P) and spliced (M) RNAs. Cells were transfected with pEGFP–GPKOW and/or pDHX16; RNA was extracted and subjected to RT–PCR using primers for an intron-containing minigene (HSPH1-i16) or an endogenous gene (BRD2, DNAJB1 or intronless SF3B5). DHX16-GN and -SL are dominant-negative mutations [24]. The% intron retention was calculated as the amount of pre-mRNA (P) over the total RNA (P+M).

Wild-type GPKOW and the GK/AA and GW/AA mutants were fused to EGFP. The expression of the fusion protein in the transfected cells was verified by immunoblots (Figure 6A) and fluorescent microscopy (Figure 6B). All three ectopically expressed proteins were localized to the nucleus (Figure 6B). To test whether GPKOW can suppress mutant DHX16-induced intron retention we co-expressed GPKOW and a mutant DHX16 with an intron-containing minigene (HSPH1-i16). Expressing the DHX16-G/N mutant (not the wild-type DHX16) resulted in accumulation of unspliced RNAs from the minigene, from endogenous genes BRD2 and DNAJB1, but did not affect intronless gene SF3B5 (Figure 6C, lane 5). Co-expressing GPKOW decreased the accumulation of unspliced RNAs–decreased the percentage of intron retention (the amount of pre-mRNA over the total RNA)–in the DHX16-G/N mutant expressing cells (Figure 6C, lane 6). Similarly, co-expressing EGFP–GPKOW also decreased the accumulation of unspliced RNAs in cells expressing another dominant-negative DHX16 mutant (DHX16-S/L; Figure 6C, compare lanes 8 and 9). Thus, GPKOW could functionally impact DHX16 mutant-induced splicing defect *in vivo*.

We also tested the two GPKOW mutants in the suppression assay and found that the GK/AA mutant was not effective in suppression (Figure 6C, lane 10), while the GW/AA mutant could suppress the DHX16 mutant defect in the RNA splicing of HSPH1 minigene and BRD2 (lane 11). It appeared that the less active mutant for *in vitro* splicing (Figure 5C) was also the less efficient mutant for *in vivo* suppression (Figure 6C). The collective results further suggested that the KOW1 domain plays an important role in the functionality of GPKOW in pre-mRNA splicing.

## DISCUSSIONS

In this report, we presented biochemical and *in vivo* evidence that GPKOW is required for human pre-mRNA splicing. Through characterization of mutants, we also showed that G-patch mediated pairwise interaction between GPKOW and DHX16 was not important for human pre-mRNA splicing, while the KOW domain next to the G-patch domain appears to be more critical.

Human GPKOW is a 55 kDa protein with one G-patch domain and two KOW motifs. A G-patch domain is typically composed of ~50 amino acids with conserved glycine at several fixed positions, and is found in RNA-processing proteins [31]. KOW motifs named after Kyrpides, Ouzounis and Woese are originally found in bacterial NusG and ribosomal proteins [32], which are 27 amino acids long with an invariant glycine at position 11. The KOW motif constitutes two β-strands that are structurally conserved. Microbial transcription modulator NusG interacts with RNA through the KOW motif [33]. A KOW domain found in Mtr4, a conserved RNA helicase, is involved in binding the RNA substrate and presenting it to the helicase core of Mtr4 [34]. The conserved positively charged lysine residue in the GK dipeptide of the KOW1 domain may interact with the negatively charged RNA backbone [33]. Thus, by changing GK residues to AA could affect the RNA interaction of GPKOW.

GPKOW is a homologue of plant MOS2, which also has a G-patch domain and two KOW motifs and is a nuclear protein critical for innate immunity in *Arabidopsis thaliana* [35]. Recently, it has been demonstrated that MOS2 is involved in miRNA maturation process in *Arabidopsis* [36]. MOS2 facilitates the recruiting of pri-miRNAs to the dicing complex by the HYL1 protein and promotes pri-miRNA processing. Genetic inactivation of a MOS2 homologue in *Caenorhabditis elegans* leads to embryonic lethality [37]. It is of great interest to investigate whether human GPKOW plays a role in microRNA processing in addition to its role in pre-mRNA splicing as shown in this and another study [22]. Moreover, GPKOW is found to interact with and be phosphorylated by protein kinase A [29]. The phosphorylation of GPKOW impairs RNA-interaction; however, it has not been investigated whether the phosphorylation would affect its splicing activity.

The G-patch domain is thought to bind single-stranded nucleic acids and to mediate protein–protein interaction. The G-patch domain of yeast Spp2 is essential for binding to yeast Prp2 and recruiting Prp2 to the spliceosome [16]. Yeast Pfa1/Ntr1 is associated with Prp43 through its G-patch domain and this interaction is important for the function of yeast Prp43 in the spliceosome [38, 39]. Our results here indicated that the G-patch of GPKOW is required for pairwise binding to DHX16, the human PRP2 homologue, although this interaction appears not to be important for human spliceosome function. GPKOW is much larger than yeast Spp2, and DHX16 is also larger than yeast Prp2. So the human proteins may have multiple, perhaps some redundant contacts with additional spliceosomal components. These potential, additional interactions may be involved in coping with a much larger intron complexity existed in multicellular organisms.

## Online data

Supplementary data
