# Supplementary material for: GPKOW is essential for pre-mRNA splicing in vitro and suppresses splicing defect caused by dominant-negative DHX16 mutation in vivo
Source: Biosci Rep. 2014 Dec 12;34(6):e00163. doi: 10.1042/BSR20140142 (PMC4266926; doi:10.1042/BSR20140142)

**Supplementary Information**

# GPKOW is essential for pre-mRNA splicing in vitro and suppresses splicing defect caused by dominant-negative DHX16 mutation in vivo

Shengbing Zang[1,2], Ting-Yu Lin[1,3], Xinji Chen[1,4], Marieta Gencheva[1], Alain N.S. Newo[1], Lixin Yang[1], Daniel Rossi[1], Jianda Hu[4], Shwu-Bin Lin[3], Aimin Huang[2*], and Ren-Jang Lin[1*]

[1]Department of Molecular and Cellular Biology, Beckman Research Institute of the City of Hope, Duarte, California
[2]Department of Pathology, School of Basic Medical Sciences, Fujian Medical University, Fuzhou, China
[3]Department of Clinical Laboratory Sciences and Medical Biotechnology, National Taiwan University, Taipei, Taiwan
[4]Fujian Institute of Hematology, Union Hospital of Fujian Medical University, Fuzhou, China

*Corresponding authors

Ren-Jang Lin, Department of Molecular and Cellular Biology, Beckman Research Institute of the City of Hope, 1500 E. Duarte Rd., Duarte, CA 91010-3000, USA
1-626-301-8286 (Phone), 1-626-301-8280 (Fax), RLin@coh.org (Email)

Aimin Huang, Department of Pathology, School of Basic Medical Sciences, Fujian Medical University, 1 Xue Yuan Rd., University Town, Fuzhou, Fujian, China
86-591-8356-9484 (Phone), 86-591-2286-2045 (Fax), aimin@mail.fjmu.edu.cn (Email)

## SUPPLEMENTARY FIGURE LEGENDS

### FIGURE S1

**Yeast two-hybrid interactions among DHX16, GPKOW, and other spliceosomal proteins.** Pair-wise interactions are linked with a line. Interactions identified by Hegele et al. are labeled in black, [1] by Aksaas et al. are in green [2], and by us in this study are in red.

### FIGURE S2

**The G-patch and the first (N-terminal) KOW domains in GPKOW homologues.** Sequence alignment of the G-patch and KOW1 domains of GPKOW from XENLA (frog), DANRE (zebrafish), mouse, and human. The conserved sequences are in bold and the dipeptide mutated in this study are in red.

### FIGURE S3

**Restoration of splicing activity in GPKOW-depleted extracts by recombinant GPKOW proteins. (A)** A denaturing gel showing the complementation of GPKOW-depleted extracts by purified $His_6$-GPKOW (WT), $His_6$-GPKOW-GK/AA (GK), and $His_6$-GPKOW- GW/AA (GW) proteins. NE, nuclear extracts; Mock, depletion using pre-immune serum. Purified proteins were added at 0.032, 0.16, and 0.8 ng/µl (depicted with a trapezoid). **(B)** Splicing assays were done similarly as (A), except the proteins were added at 0.8, 4, 20, and 100 ng/µl. Splicing efficiency was calculated by comparing spliced products with unspliced pre- mRNAs and normalized to the NE control.

## REFERENCE


1 Hegele, A., Kamburov, A., Grossmann, A., Sourlis, C., Wowro, S., Weimann, M., Will, C. L., Pena, V., Luhrmann, R. and Stelzl, U. (2012) Dynamic protein-protein interaction wiring of the human spliceosome. Mol. Cell **45**, 567-580

2 Aksaas, A. K., Larsen, A. C., Rogne, M., Rosendal, K., Kvissel, A. K. and Skalhegg, B. S. (2011) G-patch domain and KOW motifs-containing protein, GPKOW; a nuclear RNA-binding protein regulated by protein kinase A. J. Mol. Signal. **6**, 10

**Supplementary Table S1**

| *Oligonucleotide** | *Sequence (5' to 3')* |
|---|---|
| GK/AA | CACCGAGGCCTCTATGCGGCGGTGGAAGGCCTTGAT |
| GW/AA | GCTGCGGGGCATGGCCGCGAAACCTGGCGAGG |
| GPKOW F | TAAGGACTCGGATCCATGAGCGGGGAAGGGGCAGACAG |
| GPKOW R | AAGCTTGACCTCGAGTCAGGTGTTCTTGTCAAACTCCT |
| HSP/i16 F | ATAGTCGACAAAATTGGCACTCCAGTTAAAGTT |
| HSP/i16 R | ATCCCGGGCTTGATTTTTGTTTTAATTTCCTGA |
| DNAJB1 F | GAACCAAAATCACTTTCCCCAAGGAAGG |
| DNAJB1 R | AATGAGGTCCCCACGTTTCTCGGGTGT |
| BRD2 F | CAAAATTATAAAACAGCCTATGGACATG |
| BRD2 R | TTTTCCAGCGTTTGTGCCATTAGGA |
| SF3B5 F | GCACCTGCAGTCCAAGTACA |
| SF3B5 R | CCATCAAGTTGAAGCGGACT |

* F: forward; R: reverse

# SUPPLEMENTARY FIGURE S1

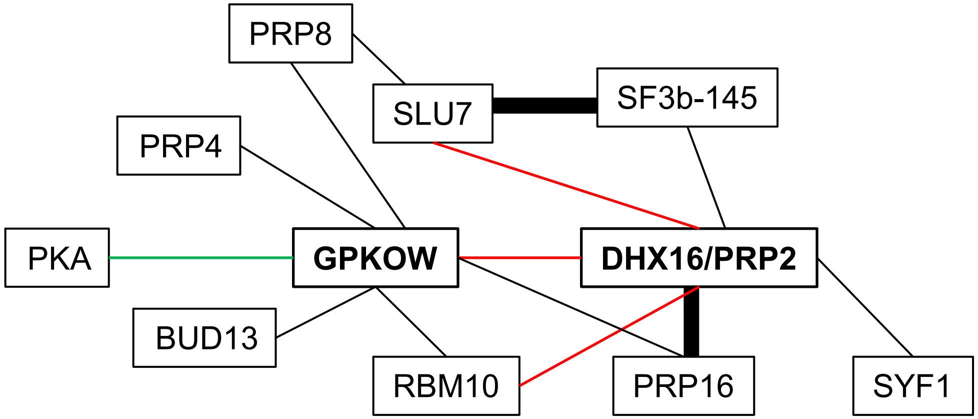

# SUPPLEMENTARY FIGURE S2

## G-Patch

Q6NU07 DV**VPVQQYGMAMLRGMGWKEGEGIGRTFKQDVKPLEQKLRPKGLGLGA**DRSALKHLEPQK 209 GPKOW_XENLA
Q90X38 ER**VPVEAYGLAMLKGMGWKQEEGIGRTFKQDVKPIEHQLRPKGLGLGA**DRSAIKDLEPGV 231 GPKOW_DANRE
Q56A08 EA**VPVEAYGLAMLRGMGWKPGKGIGNTFSQVVKPRVNSIRPKGLGLGA**NRMEAQDLASVG 219 GPKOW_MOUSE
Q92917 EA**VPVEAYGLAMLRGMGWKPGEGIGRTFNQVVKPRVNSLRPKGLGLGA**NLTEAQALTPTG 219 GPKOW_HUMAN

## KOW1

Q6NU07 PRKPLKP--GEE--PEEESKG**LGTGSAVQIQSGAYKDMYGKVEGIDPD**NSRAMITLAIGG 265 GPKOW_XENLA
Q90X38 PKRPPKP--GDEKGKEEEALV**LGPGGCVQVLAGAHKDLYGKIEGVDPD**NGRVVVKLAIGG 289 GPKOW_DANRE
Q56A08 SHHPPRPDGDRENDKEGQPQG**LMHGRAVVVLSGPYRGLYGKVEGLDPD**NVRAMVRLAVGN 279 GPKOW_MOUSE
Q92917 PSRMPRPDEEQEKDKEDQPQG**LVPGGAVVVLSGPHRGLYGKVEGLDPD**NVRAMVRLAVGS 279 GPKOW_HUMAN

# SUPPLEMENTARY FIGURE S3

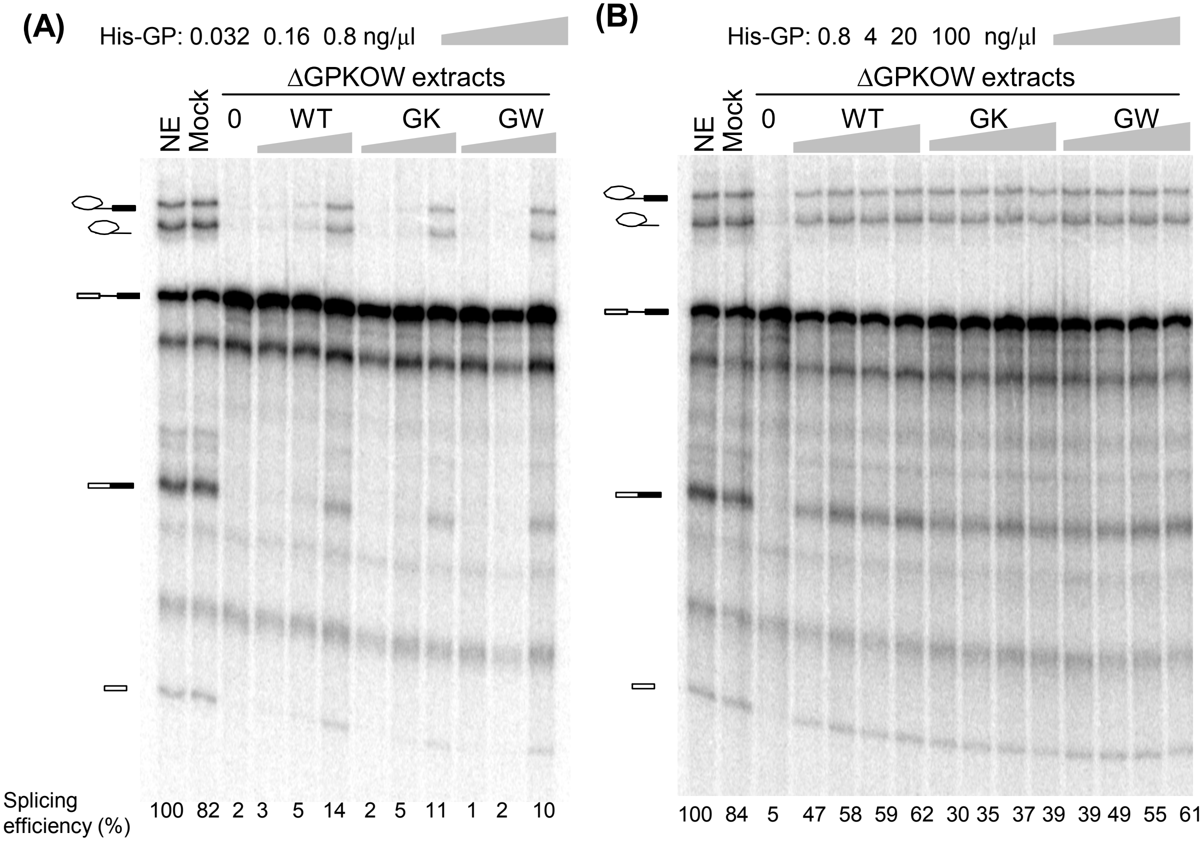

Supplement: Supplementary data [file bsr034e163ntsadd.pdf]
